# Identification of MicroRNA-21 as a Biomarker for Chemoresistance and Clinical Outcome Following Adjuvant Therapy in Resectable Pancreatic Cancer

**DOI:** 10.1371/journal.pone.0010630

**Published:** 2010-05-14

**Authors:** Jin-Hyeok Hwang, Johannes Voortman, Elisa Giovannetti, Seth M. Steinberg, Leticia G. Leon, Yong-Tae Kim, Niccola Funel, Joo Kyung Park, Min A. Kim, Gyeong Hoon Kang, Sun-Whe Kim, Marco Del Chiaro, Godefridus J. Peters, Giuseppe Giaccone

**Affiliations:** 1 Medical Oncology Branch, National Cancer Institute, National Institutes of Health, Bethesda, Maryland, United States of America; 2 Department of Internal Medicine, Seoul National University Bundang Hospital, Seongnam, Republic of Korea; 3 Department of Internal Medicine, Seoul National University College of Medicine, Seoul, Republic of Korea; 4 Department of Medical Oncology, VU University Medical Center, Amsterdam, The Netherlands; 5 Department of Internal Medicine, University of Pisa, Pisa, Italy; 6 Biostatistics and Data Management Section, National Cancer Institute, National Institutes of Health, Rockville, Maryland, United States of America; 7 Divisione di Chirurgia Generale e dei Trapianti dell'Uremico e del Diabetico, Hospital of Pisa, Pisa, Italy; 8 Department of Pathology, Seoul National University College of Medicine, Seoul, Republic of Korea; 9 Department of Surgery, Seoul National University College of Medicine, Seoul, Republic of Korea; Deutsches Krebsforschungszentrum, Germany

## Abstract

**Background:**

Pancreatic ductal adenocarcinoma (PDAC) has a dismal prognosis. The high risk of recurrence following surgical resection provides the rationale for adjuvant therapy. However, only a subset of patients benefit from adjuvant therapy. Identification of molecular markers to predict treatment outcome is therefore warranted. The aim of the present study was to evaluate whether expression of novel candidate biomarkers, including microRNAs, can predict clinical outcome in PDAC patients treated with adjuvant therapy.

**Methodology/Principal Findings:**

Formalin-fixed paraffin embedded specimens from a cohort of 82 resected Korean PDAC cases were analyzed for protein expression by immunohistochemistry and for microRNA expression using quantitative Real-Time PCR. Cox proportional hazards model analysis in the subgroup of patients treated with adjuvant therapy (N = 52) showed that lower than median miR-21 expression was associated with a significantly lower hazard ratio (HR) for death (HR = 0.316; 95%CI = 0.166–0.600; P = 0.0004) and recurrence (HR = 0.521; 95%CI = 0.280–0.967; P = 0.04). MiR-21 expression status emerged as the single most predictive biomarker for treatment outcome among all 27 biological and 9 clinicopathological factors evaluated. No significant association was detected in patients not treated with adjuvant therapy. In an independent validation cohort of 45 frozen PDAC tissues from Italian cases, all treated with adjuvant therapy, lower than median miR-21 expression was confirmed to be correlated with longer overall as well as disease-free survival. Furthermore, transfection with anti-miR-21 enhanced the chemosensitivity of PDAC cells.

**Conclusions Significance:**

Low miR-21 expression was associated with benefit from adjuvant treatment in two independent cohorts of PDAC cases, and anti-miR-21 increased anticancer drug activity *in vitro*. These data provide evidence that miR-21 may allow stratification for adjuvant therapy, and represents a new potential target for therapy in PDAC.

## Introduction

Pancreatic cancer is the fourth leading cause of cancer related death and over the last decades little improvement in survival has been observed despite extensive research efforts [1]. About 95% of exocrine pancreatic cancers are pancreatic ductal adenocarcinomas (PDAC), of which incidence has risen steadily over the last decades [1], [2]. Surgery is feasible in 15–20% of patients but, even after complete resection, prognosis remains dismal, with a 5-year survival rate lagging at 10–20% [3].

Pancreatic cancer is notoriously resistant to chemotherapy and radiotherapy. Adjuvant treatment is modestly effective and can have substantial toxicities. Therefore, the role of adjuvant therapy in resectable pancreatic cancer is still unclear, though generally thought to benefit a subset of patients [4], [5]. Being able to identify this subset would be a great advance in the management of this disease as it would allow patient stratification for adjuvant treatment [6]. Therefore, predictive markers of sensitivity to adjuvant therapy as well as new therapeutic targets are urgently needed in this disease [7], [8].

Altered expression of several factors has been associated with PDAC aggressive behaviour and prognosis. For example, among all cancers, PDAC has the highest frequency of *K-Ras* mutations, which has led to speculation regarding its application as a diagnostic as well as a prognostic marker [7]. The protein product of *K-Ras* is a GTP-binding protein mediating a number of critical cellular functions, including proliferation, cell survival and motility. However, most of the evidence, so far, suggests that *K-ras* mutations are not significantly associated with survival in pancreatic cancer patients [9], [10]. Other studies suggested the prognostic significance of altered expression of proteins involved in the Ras signaling pathway, such as Akt. Nevertheless, phosphorylated Akt expression levels were associated with both shorter and longer survival in resectable PDAC patients [11], [12]. Other markers reported as independent predictors of PDAC prognosis include p16, matrix metalloproteinase 7 (MMP-7) and vascular endothelial growth factor (VEGF) expression [13]. Still, many biological aspects governing this disease are still poorly understood and no single marker has been shown to accurately predict clinical outcome.

In recent years, it has become clear that protein expression can also be regulated by microRNAs (miRNAs) [14]. MicroRNAs are a class of small non-coding RNAs that interact with the mRNAs of coding genes to direct their posttranscriptional repression [15]. MicroRNA have been shown to be involved in oncogenesis and tumor growth, and also to play a major role in chemoresistance [16]–[19].

Furthermore, the expression of microRNAs is tissue specific, and certain cancer histotypes can be classified based on microRNA expression profiles [20], [21]. In pancreatic cancer, several miRNAs have been reported to be aberrantly expressed, including microRNAs with key roles in cancer, such as “onco(genic)-miRs”, miR-21 and miR-155, and “tumor suppressor miRs”, miR-29b and the miR-34 and Let-7 families [22]–[29]. Preclinical studies in PDAC cells showed that exogenous miR-34 overexpression was associated with reconstitution of p53-dependent tumor suppressor function in p53-deficient cells as well as inhibition of pancreatic cancer stem cell self-renewal [30]. Up-regulation of Let-7 is associated with reversal of epithelial to mesenchymal transition (EMT) in gemcitabine-resistant cells [31]. MicroRNA-29b can target de novo DNA methyltransferase 3A and 3B (DNMT3A and DNMT3B) and lead to global hypomethylation and overexpression of various tumor suppressor genes, including p16 [32]. MicroRNA-21 expression is associated with increased proliferation, invasive properties, and gemcitabine chemoresistance and a previous study showed that high miR-21 expression, as determined by in situ hybridization, was predictive of shorter survival in PDAC node-negative patients [28], [33]. MicroRNA-155 is involved in the repression of *Tumor protein 53-induced nuclear protein 1*, which is a proapoptotic stress-induced p53 target gene and a negative prognostic effect of high miR-155 expression was observed in a cohort of PDAC cases including patients with advanced disease and/or local R2-resection [34], [35].

The aim of the present study was to evaluate potential biological markers to predict outcome from adjuvant chemotherapy. Therefore, we have performed an integrative analysis of the expression of miR-21, miR-29b, miR-34a/b/c, miR-155 and let-7a-2 in a cohort of PDAC patients, with known pathological and treatment characteristics. Additionally, we have investigated expression of 20 known potential protein markers and targets for therapy, involved in PDAC progression and prognosis (See Table S1 for a complete overview of the 27 biological and 9 clinicopathological factors used in the study) [6], [13]. Since miR-21 expression status emerged as the single most predictive biomarker for treatment outcome from all factors evaluated in adjuvant-treated patients, further analysis of miR-21 expression was performed in a second cohort of 45 patients, all treated with adjuvant therapy. This independent set confirmed the significant association of miR-21 expression status with both survival and disease-free survival. In addition, the association of low miR-21 expression with benefit from adjuvant treatment was supported by *in vitro* data showing the increased chemosensitivity of PDAC cells after transfection with anti-miR-21.

## Methods

### Participants, study centers, treatment details

Two hundred forty five patients who underwent pancreatic cancer resection with the final diagnosis of pancreatic cancer were retrospectively reviewed using electronic medical records during the period 1999–2007 at Seoul National University Hospital and Seoul National University Bundang Hospital. Among them, eighty-two patients had completely resected (R0) pancreatic adenocarcinoma and were included in the study. Pathologic tumor stage was determined according to the American Joint Committee on Cancer (AJCC) Cancer Staging, 6^th^ edition [36]. Briefly, in pathologic T1-2 stages (pT1-2) tumor involvement is limited to the pancreas, tumor size being less or greater than 2 cm for stages pT1 and pT2, respectively. Pathologic T3 (pT3) stage tumors extend beyond the pancreas, without involvement of the celiac axis or superior mesenteric artery, whilst pT4 tumors are characterized by involvement of the celiac axis or the superior mesenteric artery. As for the lymph nodal stage, pathologic nodal stage 1 (pN1) indicates regional lymph node metastasis. Finally, M1 indicates presence of distant metastases. Disease stages I, IIA, IIB, III and IV indicate “pT1-2 pN0 M0”, “pT3 N0 M0”, “pT1-3 pN1 M0”, “pT4 any N M0” and “any pT any pN M1”, respectively. Patients received adjuvant chemotherapy, combined chemoradiotherapy (CCRT) or both (n = 52), or did not receive treatment (n = 27). Treatment status of three patients was unknown. Adjuvant chemotherapy and chemo-radiotherapy consisted of combinations of gemcitabine or 5-fluorouracil (5-FU). FFPE specimens were reviewed for diagnosis and tumor content at Seoul National University Hospital, as well as at National Cancer Institute, NIH, Bethesda, MD. The validation cohort was composed of frozen specimens from 45 consecutive pancreatic adenocarcinoma Italian patients diagnosed in the period 2001–2004 at the Regional Referral Center for Pancreatic Disease Treatment, University Hospital of Pisa, Pisa, Italy [37]. Adjuvant treatment consisted of gemcitabine-based combined modality treatment. Details of adjuvant regimens are listed in Table S2.

### Ethics

Patient consent and study approval was obtained from the local Institutional Review Boards according to the legal regulations of the participating countries. Informed written consent was obtained from Korean patients who were still alive at the time of the study through the human tissue bank. The study protocol was approved by the Institutional Review Board of the Human Clinical Research Center. Regarding Italian patients, written consent was obtained. The study protocol was approved by the University of Pisa Ethics Committee.

### Immunohistochemistry

The expression of 20 protein markers and targets for therapy were studied by immunohistochemistry (IHC). As reported in Table S1 we evaluated the expression levels of the following markers: vascular endothelial growth factor (VEGF), matrix metalloproteinase-2 (MMP2), matrix metalloproteinase-7 (MMP7), matrix metalloproteinase-9 (MMP9), tissue inhibitor of metalloproteinase-3 (TIMP3), epidermal growth factor receptor (EGFR), excision repair cross-complementation group 1 (ERCC1), chemokine (C-X-C motif) receptor 3 (CXCR3), chemokine (C-X-C motif) receptor 4 (CXCR4), amphiregulin, epiregulin, hepatocyte growth factor (HGF), neuropilin, insulin-like growth factor 1 receptor beta (IGF-1R), Ron β, c-Met, phosphorylated-c-Met (P-c-Met), thymidylate synthase (TS), E-cadherin, and ribonucleotide reductase subunit M1 (RRM1). Tissue microarray (TMA) sections were constructed using core tissue biopsies (diameter 2 mm) obtained from individual paraffin-embedded Korean pancreatic cancer specimens. Biopsies were included in new recipient paraffin blocks using a trephine apparatus (Superbiochips Laboratories, Seoul, Republic of Korea). Each tissue array block contained up to 50 cores and 2 array blocks were prepared during the study.

TMA sections were deparaffinized using xylene, rehydrated in alcohol and stained with specific antibodies as listed in Table S3. For ERCC1, the H-score indicates staining intensity (0 to 3) multiplied by a factor determined by the proportion of positive cells: 0 if 0% positive cells, 0.1 if 1–9%, 0.5 if 10–49%, 1 if >50% positive cells. For all other factors, positive staining meant a signal intensity equal or greater than 2 and more than 20% of positive cells.

### MicroRNA expression

RNA was isolated from FFPE sections using the RecoverAll Total Nucleic Acid Isolation kit (Ambion, Austin, TX) and from frozen tissue sections as reported previously [38]. RNA (10–100 ng) was used for expression analysis of miR-21 (Italian cohort) or miR-21, miR-29, miR-34a/b/c, let-7a-2, and miR-155 (Korean cohort) by quantitative Real-Time PCR (qRT-PCR) with TaqMan-MicroRNA assays and the 7900 HT-Fast Real-Time PCR (Applied Biosystems, Foster City, CA) using small nuclear RNA U66 or RNU43, as the endogenous normalization controls for the Korean and Italian specimens, respectively. All assays were performed in triplicate and results which did not meet methodological quality control criteria were omitted. Quantification of relative microRNA expression was performed using the delta Ct method. Expression was determined as high when the expression level was equal or above the median of the cohort and low when was below the median of the cohort.

### In vitro studies

The human PDAC cells lines BxPc3, HPAF-II, HPAC, PANC-1 and PL45 were purchased from the American Type Culture Collection (ATCC, Manassas, VA), and were cultured in RPMI-1640 media, supplemented with 10% FBS and 1% penicillin (50 IU/mL) and streptomycin (50 µg/mL) (Gibco, Gaithersburg, MD). Cells were kept at 37°C under an atmosphere of 5% CO_2_ in 75-cm^2^ tissue culture flasks (Greiner Bio-One GmbH, Frickenhausen, Germany) and harvested with trypsin-EDTA in their exponentially growing phase. RNA was extracted using a Trizol-chloroform protocol (Sigma, St. Louis, MO). RNA yields and integrity were checked by measuring optical density at 260/280 nm with a Nanodrop® spectrophotometer.

The basal expression of miR-21 was assessed by quantitative-Real-Time PCR, as described above for PDAC tissues. Quantification of miR-21 expression was performed using the delta Ct method, normalizing the Ct amplification data with RNU43. Relative miR-21 expression levels were expressed in arbitrary units (a.u.).

Cell growth inhibition by 5-FU (0.1–1000 µM), and gemcitabine (0.1–1000 nM) plus irradiation (100 cGy), was determined from three separate experiments using the SRB (sulforhodamine-B) assay, and was expressed as the percentage of control (vehicle-treated cells) absorbance, corrected for absorbance, before drug addition, as described previously [39]. For irradiation, exponentially growing cells were plated in 100-mm tissue culture dishes (Costar), allowed to attach for 24 hours, and irradiated by using a 6 MV photon linear accelerator (General Electric, Buckinghamshire, UK). After irradiation, cells were harvested and 10^4^ cells/well were plated in 96-well plates and allowed to grow for additional 48 h in drug-free medium or treated with gemcitabine, as described previously [40]. The 50% inhibitory concentration (IC_50_) of cell growth for each cell line was determined by non-linear least squares curve fitting of the dose-response curves (GraphPad PRISM version 5, Intuitive Software for Science, San Diego, CA). The effect of miR-21 on cell growth and chemosensitivity was evaluated by transfecting the cells with the antisense oligonucleotides (anti-miR-21) purchased from Ambion-Applied Biosystems (Assay ID, AM10206), at 30 nM final concentration. Cells were plated at 200,000 cells/well in 3 ml RPMI with 10% FBS and 1% antibiotics. After 24 h cells were exposed to 9 µl oligofectamine (Invitrogen, Paisley, UK) in serum-free medium, mixed for 10 minutes at room temperature, followed by the addition of 3 µl of 6.25 µM miR-21 precursor or inhibitor. Cells were also incubated with miRNA negative controls and FAM-labeled anti-mir (Ambion). After 24 hours the medium was removed from the wells and replaced with RPMI with 10% FBS, without antibiotics. Then cells were collected by trypsinization and transferred to a 96-well plate, where they were allowed to grow for additional 48 h in drug-free medium or treated with 5-FU, as described above. Additional control wells were used for RNA extraction, as described above, while the transfection efficiency with FAM-labeled anti-mir controls was evaluated with fluorescence microscopy.

### Statistical methods

Comparisons of dichotomous parameters were made between two groups using Fisher's exact test. A generalized version of Fisher's exact test was used for comparisons of age, tumor stage and differentiation grade when divided into three categories [41]. The probability of overall survival (OS) or disease-free survival (DFS) as a function of time were determined by the Kaplan-Meier method, with a log-rank test used to determine the statistical significance of the difference of Kaplan-Meier curves [42], [43] For the univariate prognostic factors analyses, cases in which patients were ultimately grouped into two categories, determined after preliminary evaluation of four categories using the quartiles of the distribution of the grouping parameter, had the p-value adjusted by multiplying the unadjusted p-value by three. This would account for the implicit testing which resulted in a decision to place patients into the two categories with a larger prognostic difference between groups. A Cox proportional hazards model analysis was performed to determine the joint association of factors initially found to have potential association with outcome in the univariate analyses (evaluating in this *final model* only those parameters with unadjusted p<0.10 from a log-rank test) [44]. All p-values are two-tailed, and except as noted above, are presented without adjustment for multiple comparisons.

All *in vitro* experiments were performed in triplicate and repeated at least two times. Data was expressed as mean values±SE and analyzed by Student's t test and/or Mann Whitney test. Statistical significance was set at p<0.05.

## Results

### Characteristics of the patients

Table 1 summarizes clinicopathological characteristics. In the Korean patients, median OS and DFS were 18.1 and 9.1 months, respectively. In this cohort OS was significantly longer for patients treated with adjuvant chemotherapy and/or CCRT (n = 52): median survival was 21.3 vs. 14.7 months for patients treated vs. not treated with adjuvant therapy (n = 27); p = 0.017. See Figure S1 for Kaplan-Meier plots.

**Table 1:** Clinical characteristics of pancreatic ductal adenocarcinoma (PDAC) patients.

Characteristic	Subcategory	Korean cohort, n	Italian cohort, n
**No. patients**	N/A	82	45
**Age, years**	≤64	41	24
	>64	41	21
**Sex**	Male	53	17
	Female	29	28
**p-AJCC stage** *	I	0	0
	IIA	31	7
	IIB	50	26
	III	1	8
	IV	0	4
**Tumor size** **	<15 mm	3	Na
	15–20 mm	10	Na
	>20 mm	69	Na
**Lymph node**	negative	29	8
	positive	51	37
	unknown	2	0
**Differentiation grade**	Well	6	5
	Moderate	68	20
	Poor	8	19
	Unknown	0	1
**Angiolymphatic invasion**	No	43	Na
	Yes	39	Na
**Venous invasion**	No	65	Na
	Yes	17	Na
**Vascular invasion (venous and arterial)**	No	Na	31
	Yes	Na	14
**Perineural invasion**	No	23	32
	Yes	59	13
**Adjuvant therapy**	Yes	52	45
	No	27	0
	Unknown	3	0

Abbreviations: N/A: not applicable; Na: not available.

*pAJCC pathologic tumor stage was determined according to the American Joint Committee on Cancer (AJCC) Cancer Staging, 6^th^ edition.

**Since there were no cases of tumor size<10 mm, tumor size was categorized in the following 3 groups: <15 mm, 15–20 mm and >20 mm.

### Expression analysis results

Expression of miR-21, miR-34a, miR-155 and let-7a was detectable in all 82 cases, and expression of miR-29b, miR-34b, and miR-34c in 81, 73 and 80 cases, respectively. In agreement with previous studies showing that miR-34b and miR-34c share the same transcription promoter [45], expression values of these miRNAs were correlated (data not shown). IHC results were available for neuropilin and MMP-9 in 80 cases, for amphiregulin, HGF, EGFR and TIMP3 in 79 cases, for Ron, CXCR-3, e-cadherin, RRM1, IGF-1R, TS, VEGF, MMP-2 and MMP-7 in 78 cases, for CXCR-4 in 77 cases, for c-Met and P-c-Met in 76 cases, for ERCC1 in 75 cases and for epiregulin in 73 cases.

As described in the Methods section, for each protein we scored the staining intensity and the percentage of positively stained cells. In Figure S2, representative images of immunohistochemical staining for P-c-Met are shown. Table 2 summarizes the results of immunohistochemical staining for all the studied proteins.

**Table 2:** Tissue microarray immunohistochemistry results Korean pancreatic adenocarcinoma patients.

Proteins	Expression	Total, n (%)
**Amphiregulin**	Negative	27 (36%)
	Positive	49 (64%)
**Epiregulin**	Negative	22 (31%)
	Positive	49 (69%)
**Ron β**	Negative	18 (24%)
	Positive	57 (76%)
**HGF**	Negative	29 (38%)
	Positive	47 (62%)
**CXCR3**	Negative	64 (85%)
	Positive	11 (15%)
**CXCR4**	Negative	11 (15%)
	Positive	63 (85%)
**E-cadherin**	Negative	1 (1%)
	Positive	74 (99%)
**RRM1**	Negative	62 (83%)
	Positive	13 (17%)
**ERCC1**	Negative	46 (64%)
	Positive	26 (36%)
**ERCC1 (H-score)**	Negative	18 (25%)
	Positive	54 (75%)
**TS**	Negative	71 (95%)
	Positive	4 (5%)
**EGFR**	Negative	59 (78%)
	Positive	17 (22%)
**IGF-1R**	Negative	57 (76%)
	Positive	18 (24%)
**Neurophilin**	Negative	27 (35%)
	Positive	50 (65%)
**VEGF**	Negative	25 (33%)
	Positive	50 (67%)
**c-Met**	Negative	56 (77%)
	Positive	17 (23%)
**phosporylated-c-Met**	Negative	40 (55%)
	Positive	33 (45%)
**MMP2**	Negative	26 (35%)
	Positive	49 (65%)
**MMP7**	Negative	21 (28%)
	Positive	54 (72%)
**MMP9**	Negative	7 (9%)
	Positive	70 (91%)
**TIMP3**	Negative	28 (37%)
	Positive	47 (63%)

Abbreviations: Chemokine (C-X-C motif) receptor 3 (CXCR3), chemokine (C-X-C motif) receptor 4 (CXCR4), epidermal growth factor receptor (EGFR), excision repair cross-complementation group 1 (ERCC1), hepatocyte growth factor (HGF), insulin-like growth factor 1 receptor beta (IGF-1R), matrix metalloproteinase-2 (MMP2), matrix metalloproteinase-7 (MMP7), matrix metalloproteinase-9 (MMP9), ribonucleotide reductase subunit M1 (RRM1), thymidylate synthase (TS), tissue inhibitor of metalloproteinase-3 (TIMP3) and vascular endothelial growth factor (VEGF).

### Univariate analysis

**Figure 1:**
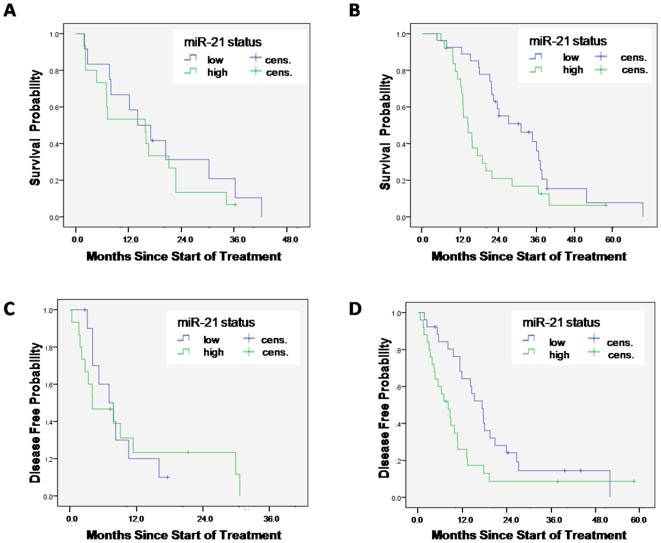
Survival analysis of adjuvant treated and not adjuvant treated patients. Overall survival according to miR-21 status in (A) not adjuvant treated patients and (B) adjuvant treated patients. Disease-free survival according to miR-21 status in (C) not adjuvant treated patients and (D) adjuvant treated patients. *Cens.: censored*.

A total of 36 clinicopathological, microRNA and protein variables were evaluated for their association with OS or DFS in the Korean cohort. Based on univariate analysis, a set of 10 and 12 parameters were considered for evaluation in the Cox models for OS and DFS, respectively (see Table S4). Selected parameters were also tested according to adjuvant treatment status (Table S4). Additionally, for any parameter, the population was divided into 4 groups for comparison: (1) negative parameter, no adjuvant treatment vs. (2) negative parameter, adjuvant treatment vs. (3) positive parameter, no adjuvant treatment vs. (4) positive parameter, adjuvant treatment. Table S4 only lists combinations with an associated p-value <0.10. Notably, a strong interaction of miR-21 expression status and adjuvant treatment status for OS and DFS was demonstrated. Low miR-21 expression was associated with longer OS and DFS in the adjuvant treated patients, with p-values of 0.016 and 0.02, respectively, but not in patients not treated with adjuvant therapy (p-values of 0.49 and 0.93, respectively). This differential association between miR-21 and treatment status was supported by comparing the subgroup of low miR-21, adjuvant treated patients vs. remaining patients. The group of patients with low miR-21 who were treated with adjuvant treatment had median OS and DFS of 27.7 and 16.2 months, respectively. Remaining patients had median OS of 14.3 and DFS of 7.0 months, with p-values of 0.002 for OS, and 0.0095 for DFS. See also Figure 1 and Figure S3.

### Cox models for overall survival and disease-free survival

Using a backward selection algorithm, a Cox model was constructed containing parameters that were found to be jointly associated with OS. A second model was identified for prognostic determination of DFS (Tables 3 and 4). From multivariate analysis among all patients, as the most significantly associated parameter for both OS and DFS, patients with low miR-21 who were treated with adjuvant therapy were shown to have a significantly lower hazard ratio (HR) for death (0.443, 95%CI: 0.263–0.748; p-value, 0.002) and for recurrence (0.358, 95%CI: 0.188–0.682; p-value: 0.002) compared to all remaining patients. In contrast, in patients not treated with adjuvant therapy, lower than median miR-21 expression was not associated with a significantly different HR for death or recurrence of 0.880, p-values 0.76 and 0.30, respectively.

**Table 3:** Multivariate analysis of all patients: overall survival.

Comparison	Hazard ratio for death	95% CI	p-value
(Adjuvant treated, miR-21 negative) vs. rest	0.443	0.263–0.748	0.0023
Angiolymphatic invasion: positive vs. negative	1.769	1.091–2.868	0.0208

**Table 4:** Multivariate analysis of all patients: disease-free survival.

Comparison	Hazard ratio for recurrence	95% CI	p-value
(Adjuvant treated, miR-21 negative) vs. rest	0.358	0.188–0.682	0.0018
Angiolymphatic invasion: positive vs. negative	1.923	1.106–3.344	0.0206
Amphiregulin status: positive vs. negative	0.512	0.276–0.951	0.0342
(Not adjuvant treated, miR-34a positive) vs. rest	7.375	2.315–23.491	0.0007
Adjuvant treated vs. rest	0.419	0.196–0.894	0.0244
(Adjuvant treated, pN negative) vs. rest	0.310	0.152–0.632	0.0013

### Comparison of covariates according to adjuvant treated vs. untreated patients

Since cases were not randomized for treatment in this study, we wanted to determine if there were significant differences in the covariate distribution between the patients treated and not treated with adjuvant therapy (Table S5). The only significant difference was a lower median age in the adjuvant treated group at 61 years (range: 45–75 years) vs. 66 years (range: 49–83 years) in the untreated group (p-value: 0.002, Wilcoxon two-sample test). See Table S6 for differential protein expression. Additionally, we constructed separate Cox models for patients treated with adjuvant therapy vs. patients not treated with adjuvant therapy. First, we did a univariate analysis in both groups (Tables S7 and S8). Cox model analysis demonstrates that only in the subgroup of adjuvant treated patients low miR-21 status was associated with a significantly lower HR for death and recurrence (Table 5, Table 6, Table 7 and Table 8). In the subgroup of not adjuvant treated patients, positive angiolymphatic invasion and expression of miR-34a above the median were associated with increased HR for death and recurrence, respectively, while miR-21 did not have a sufficiently low p-value for inclusion in the final model.

**Table 5:** Multivariate analysis of adjuvant treated patients: overall survival.

Comparison	Hazard ratio for death	95% CI	p-value
(Adjuvant treated, miR-21 negative) vs. rest	0.316	0.166–0.600	0.0004
CXCR3 status: positive vs. negative	4.177	1.775–9.831	0.0011
AJCC stage: IIB vs. IIA	2.092	1.078–4.058	0.0290

**Table 6:** Multivariate analysis of adjuvant treated patients: disease-free.

Comparison	Hazard ratio for recurrence	95% CI	p-value
(Adjuvant treated, miR-21 negative) vs. rest	0.521	0.280–0.967	0.0387
pN status: positive vs. negative	1.968	1.026–3.775	0.0416

**Table 7:** Multivariate analysis of patients not treated with adjuvant therapy: overall survival.

Comparison	Hazard ratio for death	95% CI	p-value
Angiolymphatic invasion: positive vs. negative	3.452	1.306–9.125	0.0125

**Table 8:** Multivariate analysis of patients not treated with adjuvant therapy: disease-free survival.

Comparison	Hazard ratio for recurrence	95% CI	p-value
miR-34a status: positive vs. negative	4.435	1.604–12.263	0.0041

### Comparison of covariates according to miR-21 expression

Distribution of clinicopathological and IHC covariates was compared between low miR-21 and high miR-21 patients, as reported in Table 9 and Table S9. Median age in the low miR-21 group was 63 years (range: 45–83 years), not significantly different from the high miR-21 group, median age 64 years (range: 46–78 years), p-value: 0.94. However, there was a trend towards a more advanced AJCC stage (IIa vs IIb) in the high miR-21 expression group (p-value: 0.07). Similarly, there were more cases with poor differentiation grade in the high miR-21 group, although this group also contained more cases with well-differentiated tumors (Table 5). We could not confirm a previously reported association of miR-21 and TIMP3 expression [46], while we found that P-c-Met and VEGF expression was significantly more common in miR-21 high cases (Table S9).

**Table 9:** Korean cohort: association of miR-21 expression with clinicopathological covariates.

Characteristic	Subcategory	Low miR-21, n (%)	High miR-21, n (%)	Total, n (%)	p-value
**Sex**	Male	26 (63%)	27 (66%)	53 (65%)	1.00
	Female	15 (37%)	14 (34%)	29 (35%)	
**Age, years**	<55	6 (15%)	4 (10%)	10 (12%)	0.65
	55-64	17 (41%)	21 (51%)	38 (46%)	
	>64	18 (44%)	16 (39%)	34 (41%)	
**p-AJCC stage** *	IIa	20 (49%)	11 (28%)	31 (38%)	0.07
	IIb	21 (51%)	29 (73%)	50 (62%)	
**Tumor size** **	<15 mm	2 (5%)	1 (2%)	3 (4%)	1.00
	15–20 mm	5 (12%)	5 (12%)	10 (12%)	
	>20 mm	34 (83%)	35 (85%)	69 (84%)	
**pN stage**	0	18 (44%)	11 (27%	29 (35%)	0.16
	1	22 (54%)	29 (71%)	51 (62%)	
	unknown	1 (2%)	1 (2%)	2 (2%)	
**Differentiation grade**	Well	1 (2%)	5 (12%)	6 (7%)	0.02
	Moderate	37 (90%)	29 (71%)	66 (80%)	
	Poor/undifferentiated	1 (2%)	7 (17%)	8 (10%)	
	unknown	2 (5%)	0 (0%)	2 (2%)	
**Angiolymphatic invasion**	No	24 (59%)	19 (46%)	43 (52%)	0.38
	Yes	17 (41%)	22 (54%)	39 (48%)	
**Venous invasion**	No	31 (76%)	34 (83%)	65 (79%)	0.59
	Yes	10 (24%)	7 (17%)	17 (21%)	
**Perineural invasion**	No	14 (34%)	9 (22%)	23 (28%)	0.33
	Yes	27 (66%)	32 (78%)	59 (72%)	

*pAJCC pathologic tumor stage was determined according to the American Joint Committee on Cancer (AJCC) Cancer Staging, 6^th^ edition.

**Since there were no cases of tumor size<10 mm, tumor size was categorized in the following 3 groups: <15 mm, 15–20 mm and >20 mm.

As high miR-34a expression was demonstrated to be associated with decreased DFS we have additionally compared distribution of clinicopathological and IHC covariates between low miR-34a and high miR-34a patients. High expression of miR-34a was strongly associated with high expression of phospho-c-Met (p-value: 1x10E-6). Additionally, high miR-34a expression was associated with low expression of HGF (p-value: 0.01) as well as high expression of VEGF (p-value: 0.01). Finally, low miR-21 expression was more frequently observed in miR-34a low cases (p-value: 3x10E-8).

### High miR-21 expression shows association with increased distant recurrence rate

Next, we compared the rate of local vs. distant recurrence of disease in the low vs. high expression group of miR-21. In the high miR-21 expression group 25 out of 41 patients (61%) had recurrent disease at a distant site which was significantly different from the low miR-21 expression group in which only 12 out of 38 patients (32%; with 3 patients lost for follow-up) experiencing distant recurrence (p-value: 0.013). For comparison, in the group that received adjuvant therapy 25 out of 51 patients (49%) had recurrent disease. This was not significantly different from the group not treated with adjuvant therapy with 11 out of 26 patients (42%) experienced distant recurrence (p-value: 0.63; with 5/82 patients missing due to insufficient data).

Although not statistically significant, high miR-21 expression also showed a trend towards higher stage and positive lymph node status (Table 5).

### Validation cohort

**Figure 2:**
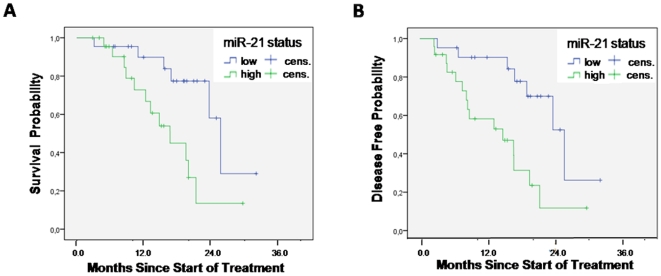
Survival analysis in adjuvant treated patients from the Italian cohort. (A) Overall survival according to miR-21 status and (B) disease-free survival status according to miR-21 status. *Cens.: censored*.

A series of 45 resected pancreatic adenocarcinoma patients, all treated with adjuvant gemcitabine, was used to validate the predictive role of miR-21 expression in Caucasians. Median OS and DFS were 20.4 and 18.7 months, respectively. See Table 1 for clinicopathological parameters (Italian cohort). MiR-21 expression was detectable in all specimens. Univariate analysis showed a trend towards significant association between poor tumor differentiation and shorter OS (p-value: 0.07), while the occurrence of neural infiltration was marginally associated with significantly shorter DFS (p-value: 0.047), but not OS (p-value: 0.90), as reported in Table S10. Age, gender, stage, lymph node, and vascular infiltration were not associated with outcome. Patients with high miR-21 expression had a significantly shorter OS, i.e. median 15.6 compared to 24.4 months in patients with miR-21 expression level inferior to median (p-value: 0.006). The median DFS of patients with high miR-21 expression was 14.0 months, compared to 23.8 months in patients with the low miR-21 expression (p-value: 0.0042). See Figure 2 for Kaplan-Meier curves. Cox models were constructed which confirmed the association of miR-21 expression status and survival duration, with high miR-21 expression, as the only remaining factor, associated with an increased HR for death at 3.538 (95%CI: 1.415–8.849; p-value: 0.007) and recurrence at 4.008 (95%CI: 1.385–11.595; p-value: 0.01). Pooled analysis of the two cohorts confirmed the significant predictive importance of miR-21 expression status, as reported in Figures S4 and S5.

### MiR-21 and antiproliferative effects of 5-FU in PDAC cells

**Figure 3:**
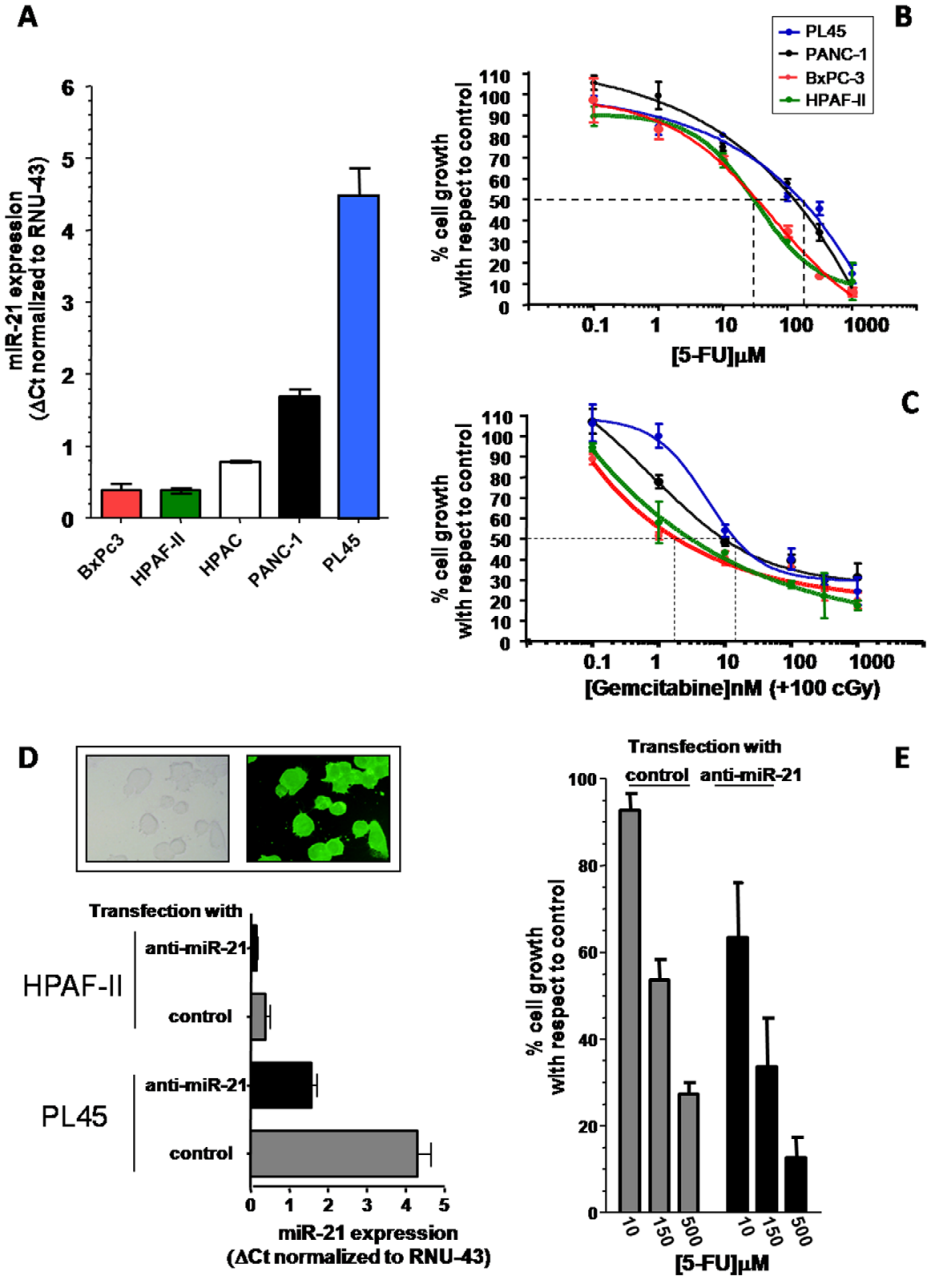
In vitro studies validating the role of miR-21 in PDAC chemosensitivity. (A) MiR-21 expression in 5 PDAC cell lines. Expression was determined by quantitative PCR, using the delta Ct method with RNU-43 as reference and the values are in a.u.. (B–C) Representative curves of growth inhibitory effects of 5-FU (B) and gemcitabine plus radiotherapy (C), 48-hour drug exposure. (D). Cells were seeded at 10^4^/well and the anti-proliferative effects were evaluated using the SRB assay, as described in the methods. The mean IC_50_ values for 5-FU (48 h continuous exposure) were as follows: 36.3 µM (BxPC-3), 30.9 µM (HPAF-II), 138.4 µM (PANC-1) and 174.2 µM (PL45); while the IC50 values for gemcitabine (in cells pre-treated with 100 cGray) were 2.1 nM (BxPC-3), 3.4 nM (HPAF-II), 10.2 nM (PANC-1) and 11.4 nM (PL45). (D) MiR-21 expression in HPAF-II and PL45 cells transfected with negative controls or with anti-miR-21 oligos. Transfection efficiency was evaluated by fluorescence microscopy as shown in the pictures in the upper panel, original magnification, x40 (E) Modulation of 5-FU antiproliferative effects in PL45 cells transfected with anti-miR-21 in comparison with control transfected cells. *Columns*, mean values obtained from three independent experiments; *bars*, SE, *dashed lines*, concentrations corresponding to 50% inhibition of cell growth with respect to control, i.e. IC_50_ values.

Expression of miR-21 was detectable in all PDAC cell lines, ranging from 4.5 in PL45 cells to 1.5 a.u. in the BxPC-3 cells (Figure 3A). A dose-dependent inhibition of cell growth was observed after both 5-FU, and gemcitabine plus radiotherapy treatment, as shown in Figure 3B-C. In particular, 5-FU treatment resulted in a modest inhibition of cell growth in PANC-1 and PL45 cells, with IC_50_s of 138.4±23.4 µM and 174.2±31.1 µM, respectively. Among the studied cell lines, BxPC3 and HPAF-II were the most sensitive to gemcitabine and 5-FU, respectively, while PL45 was the most resistant to both 5-FU and gemcitabine plus radiotherapy.

Although the small number of cell lines used in this study precluded the assessment of the predictive value of miR-21 expression as a validated determinant of chemosensitivity, the cell lines with low miR-21 expression (BxPC-3 and HPAF-II) had a significantly lower IC_50_ than the cell lines with high miR-21 expression (PANC-1 and PL45), p-value: 0.0079 (Mann-Whitney test).

To further explore this potential association, relatively sensitive (HPAF-II), and resistant (PL45) cells were transfected with a miR-21 specific antisense inhibitor. Transfection efficiency was evaluated by analysis of fluorescent microscope images of cells 24 hours post transfection with a specific FAM-dye precursor or antisense anti-mir oligonucleotides (Figure 3D, picture). There was at least 80% transfection efficiency, for both cell lines, with >70% cell viability. Furthermore, we assessed miR-21 expression by quantitative Real-Time PCR in the transfected cells, showing a 3 and 2.5-fold decrease of miR-21 expression in PL45 and HPAF-II cells, respectively (Figure 3D, bar graph). In order to evaluate the modulation of 5-FU anti-proliferative effects, we studied whether co-treatment of anti-miR-21 and 5-FU in PL45 cells would result in increased sensitivity to 5-FU. For this experiment, the selected cell lines were pretreated with anti-miR-21 for 24 hours, followed by 5-FU for an additional 48 hours. As shown in Figure 3E the transfection with anti-miR-21 resulted in an increased activity of 5-FU, with a reduction of 5-FU IC_50_ values from 174.2±31.1 to 62.5±15.9 µM in PL45 cells and from 30.9±3.4 to 11.3±2.1 µM in HPAF-II cells.

## Discussion

In the present study 27 biological (20 proteins and 7 microRNAs) and 9 clinicopathological factors were jointly assessed in order to determine biomarkers for clinical outcome in patients treated or not treated with adjuvant therapy following surgical resection of PDAC. Of these 36 covariates analyzed, miR-21 expression was the only factor consistently associated with OS and DFS in all Cox model analyses when evaluated according to treatment status in an initial series of 82 Korean patients. Adjuvant treated patients with low miR-21 expression were shown to have a favorable outcome compared to patients with high miR-21 expression.

Our findings are in agreement with a previous study which showed that miR-21 overexpression, as determined by in situ hybridization, was predictive of shorter survival only in PDAC node-negative patients. However, the subset of node-negative patients was small (n = 17) and no data were available on patient treatment or other clinicopathological characteristics [28]. Our study extended the analysis to patients receiving adjuvant therapy and compared these patients with not treated patients. Furthermore, we evaluated a wide variety of clinicopathological and biological factors suggesting that miR-21 is the most predictive marker for survival of any of these factors in adjuvant-treated patients.

According to the final results of the recently reported CONKO-001 and ESPAC-3 trials, adjuvant chemotherapy increased DFS and OS duration [47]–[49]. Still, the most effective adjuvant regimen and the role of radiotherapy remain unclear, and identification of predictive factors for survival is critical to maximize therapeutic efficacy in selected patients.

The expanding knowledge of the molecular pathogenesis of cancer is providing new targets for disease characterization, which might also be used as new markers to select patients for better clinical management. In particular, there is a growing number of studies on miRNAs, which are classified as oncogenes or tumor-suppressor genes and have a pivotal role in progression and prognosis of different tumors [16]. A specific miRNA can affect simultaneously the expression of proteins involved in multiple cellular pathways, potentially serving as better therapeutic target or biomarker for clinical outcome than single proteins. Furthermore, technological advances have made it possible to reliably determine miRNA expression using FFPE tissues, and previous studies showed that miRNA expression in FFPE correlated with expression in matched fresh/frozen tissues [50]–[53]. Accordingly, in the present study miRNAs were successfully extracted and evaluated from both FFPE and frozen laser microdissected specimens.

The miRNAs currently analyzed were selected from the comparison of miRNA expression patterns in normal and tumoral pancreatic tissues [29], as well as from the results of several preclinical studies, suggesting their role in tumor progression and sensitivity [13], [31], [33], [34]. In particular, the transfection with miR-21 precursor reduced gemcitabine sensitivity of PANC-1 cells [33], while antisense inhibition of miR-21 led to cell cycle arrest, induced apoptosis and sensitized the effects of gemcitabine in HS766T cells [54], suggesting a key role of miR-21 in modulating the response to this specific drug in PDAC cells. However, miR-21 expression was correlated with resistance to several anticancer agents in different models [55]–[58]. The studies of Meng *et al.* showed a correlation of miR-21 expression and gemcitabine-induced apoptosis and modulation of PTEN and associated pathways, thus affecting phenotypic characteristics such as cell growth, migration, and invasion in cholangiocarcinoma and hepatocellular carcinoma *in vitro* and *in vivo* [55], [56]. More recently, Li *et al.* found that the repression of miR-21 expression sensitizes glioblastoma cells to VM-26 treatment via leucine rich repeat interacting protein (LRRFIP1)-mediated inhibition of NF-κB signaling, a principal mechanism of tumor chemoresistance [18]. Moreover, combined suppression of miR-21 with S-TRAIL in glioma cells leads to a synergistic cytotoxicity and increased caspase activity, which was associated with reduction of tumor growth both *in vitro* and *in vivo* [59]. Inhibition of miR-21 expression has also been shown to sensitize MCF-7 cells to topotecan by inducing an increased apoptotic response, partly caused by downregulation of the anti-apoptotic protein Bcl-2 [60].

The patients of the Korean cohort enrolled in this study were treated with various gemcitabine or 5-FU containing adjuvant regimens, suggesting that miR-21 expression can affect outcome of both gemcitabine and 5-FU-based treatment. Accordingly, the higher expression of miR-21 was detected in PDAC cells with the higher IC_50_ values for 5-FU, while miR-21 suppression with a specific anti-miR significantly increased the antiproliferative effects of 5-FU. To our knowledge, this is the first study showing a correlation between miR-21 expression and 5-FU activity and these data might explain the results of a previous clinical study, showing that high miR-21 expression was associated with poor outcome in colon cancer patients treated with adjuvant 5-FU-based chemotherapy [61].

In the present study, high miR-21 expression was also associated with an increased distant recurrence rate, suggesting a role in the metastatic behavior of pancreatic adenocarcinoma. Accordingly, high miR-21 expression levels correlated with increased proliferation and occurrence of liver metastasis in previous studies in PDAC cells [33] and in pancreatic endocrine tumors [62]. However, as shown by the analysis in the subgroup of patients not treated with adjuvant therapy, low miR-21 expression on its own was not associated with prolonged OS or DFS, negating its role as a purely prognostic factor.

Several other parameters were associated with OS or DFS according to adjuvant treatment status, including angiolymphatic invasion, CXCR3 and miR-34a. However, in the Cox model analyses, none of these markers were found to be associated with both OS and DFS in the adjuvant treated patient subgroup and/or the untreated group. Positive angiolymphatic invasion, as determined on the surgical specimen, was associated with worse OS and DFS in the total population and with worse OS in the patient subgroup not treated with adjuvant therapy, as reported previously [63]. However, in the treated subgroup, angiolymphatic invasion was not a significant factor at the univariate analysis. In adjuvant treated patients positive CXCR3 expression and advanced stage were also associated with worse treatment outcome in terms of OS. Recent studies suggested that the CX3CR1 receptor may be involved in PDAC neurotropism and is a relevant and independent risk factor to predict an early local tumor relapse in resected patients, but further studies are needed to unravel the complex network of chemokines and their receptors in the pancreatic cancer microenvironment [64]. High miR-34a expression was associated with decreased DFS at the multivariate analysis in the not adjuvant treated patients. Furthermore, we found a significant correlation between high miR-34a expression and high VEGF and p-c-Met levels, which have been correlated with increased microvessel density, tumor metastatic potential, local disease progression and chemoresistance in a variety of malignancies, including PDAC [65], [66]. However, high expression levels of VEGF and p-c-Met were also associated with high expression of miR-21 and the comparison between miR-21 (low vs. high) with miR-34a (low vs. high) expression showed a significant association, likely explaining why both these two miRNAs did not typically end up in the same Cox model.

Although miR-21 expression was shown to be the single most important predictive factor from 36 covariates tested, it is crucial to validate findings by follow-up prospective studies in independent cohorts with controlled treatment regimens. In the present study miR-21 was confirmed to be a biomarker for treatment outcome in an independent patient cohort. This second cohort was added only after the end of the first study, and we are aware of the limitations of our comparison. Interestingly, these patients had different ethnicity (Caucasian vs. Asian), specimens (frozen tissues vs. FFPE), treatment (gemcitabine + radiotherapy vs. gemcitabine or fluoropyrimidines) and endogenous controls in the PCR reactions (RNU43 vs. U66), suggesting that miR-21 might be used as effective biomarker for different populations/treatments, and when different types of specimens/PCR reagents are available.

Concluding, low miR-21 expression was associated with increased survival following adjuvant treatment in two independent cohorts of PDAC cases, and anti-miR-21 increased anticancer drug activity *in vitro*. These data provide evidence that miR-21 may allow stratification for adjuvant therapy, thus offering a potential new biomarker for treatment selection and personalized therapy. Furthers studies are warranted.

## Supporting Information

Figure S1 Survival curves of Korean pancreatic adenocarcinoma patients. (A) overall survival and (B) disease-free survival, in total study population. (C) overall survival, by pAJCC stage. (D) overall survival, by treatment status. Cens.: censored.
(5.08 MB TIF)

Figure S2 Representative images of two pancreatic adenocarcinoma cases from the Korean cohort stained by immunohistochemistry for phosphorylated c-Met (p-c-Met), according to the methodology and scoring algorithm as described in the Methods section. A, example of a case with a positive staining pattern for phosphorylated c-Met; B, example of a case with a negative staining pattern for phosphorylated c-Met.
(3.82 MB TIF)

Figure S3 Survival curves according to miR-21 expression and treatment status (A) overall survival and (B) progression-free survival, according to miR-21 status combined with adjuvant treatment status. (C) overall survival and (D) disease-free survival, comparing low miR-21 patients who received adjuvant chemotherapy to rest. Abbreviations used: “miR-21 high/adj”: high miR-21, having received adjuvant treatment; “miR-21 low/adj”: low miR-21 expression, having received adjuvant treatment; “miR-21 high/no adj”: high miR-21, no adjuvant treatment; “miR-21 low/no adj”: low miR-21, no adjuvant treatment. Cens.: censored.
(5.10 MB TIF)

Figure S4 Pooled analysis Korean and Italian cohorts: disease-free survival curves according to miR-21 expression and treatment status.
(0.19 MB TIF)

Figure S5 Pooled analysis Korean and Italian cohorts: overall survival curves according to miR-21 expression and treatment status.
(0.19 MB TIF)

Table S1 Clinicopathological and biological factors analyzed.
(0.05 MB DOC)

Table S2 Adjuvant therapy regimens.
(0.05 MB DOC)

Table S3 Antibodies used for immunohistochemistry.
(0.04 MB DOC)

Table S4 Univariate analysis Korean cohort.
(0.07 MB DOC)

Table S5 Korean cohort: clinicopathological covariates according to treatment status.
(0.07 MB DOC)

Table S6 Korean cohort: immunohistochemistry covariates according to treatment status.
(0.10 MB DOC)

Table S7 Korean cohort: univariate analysis in adjuvant treated patients.
(0.04 MB DOC)

Table S8 Korean cohort: univariate analysis in not adjuvant treated patients Korean cohort.
(0.04 MB DOC)

Table S9 Korean cohort: association miR-21 and immunohistochemistry covariates.
(0.09 MB DOC)

Table S10 Italian cohort: univariate analysis in adjuvant treated patients.
(0.04 MB DOC)
