# Supplementary figures and images for: Identification of MicroRNA-21 as a Biomarker for Chemoresistance and Clinical Outcome Following Adjuvant Therapy in Resectable Pancreatic Cancer
Source: PLoS One. 2010 May 14;5(5):e10630. doi: 10.1371/journal.pone.0010630 (PMC2871055; doi:10.1371/journal.pone.0010630)

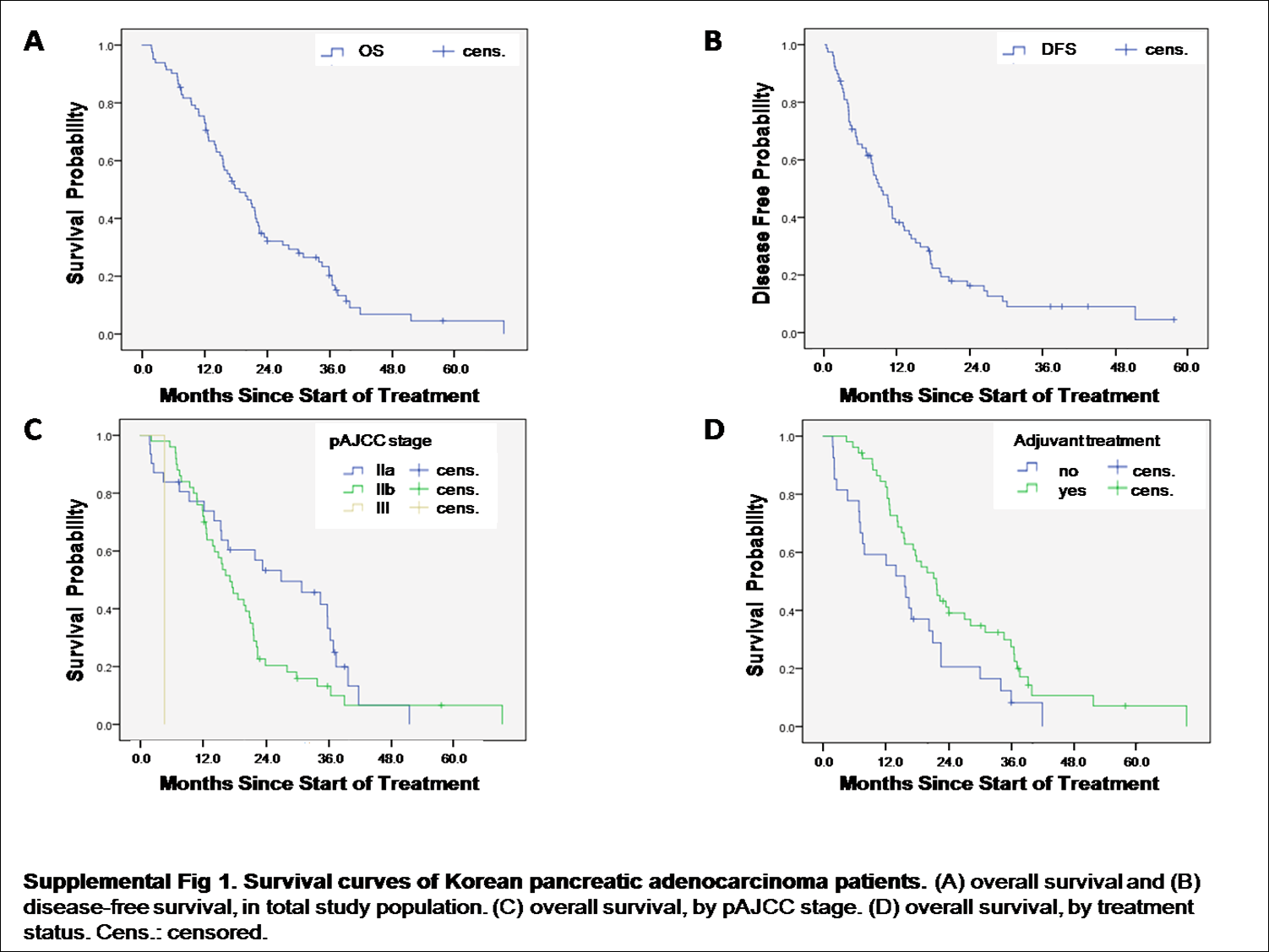

Supplement: Figure S1 — Survival curves of Korean pancreatic adenocarcinoma patients. (A) overall survival and (B) disease-free survival, in total study population. (C) overall survival, by pAJCC stage. (D) overall survival, by treatment status. Cens.: censored. (5.08 MB TIF) [file pone.0010630.s001.tif]

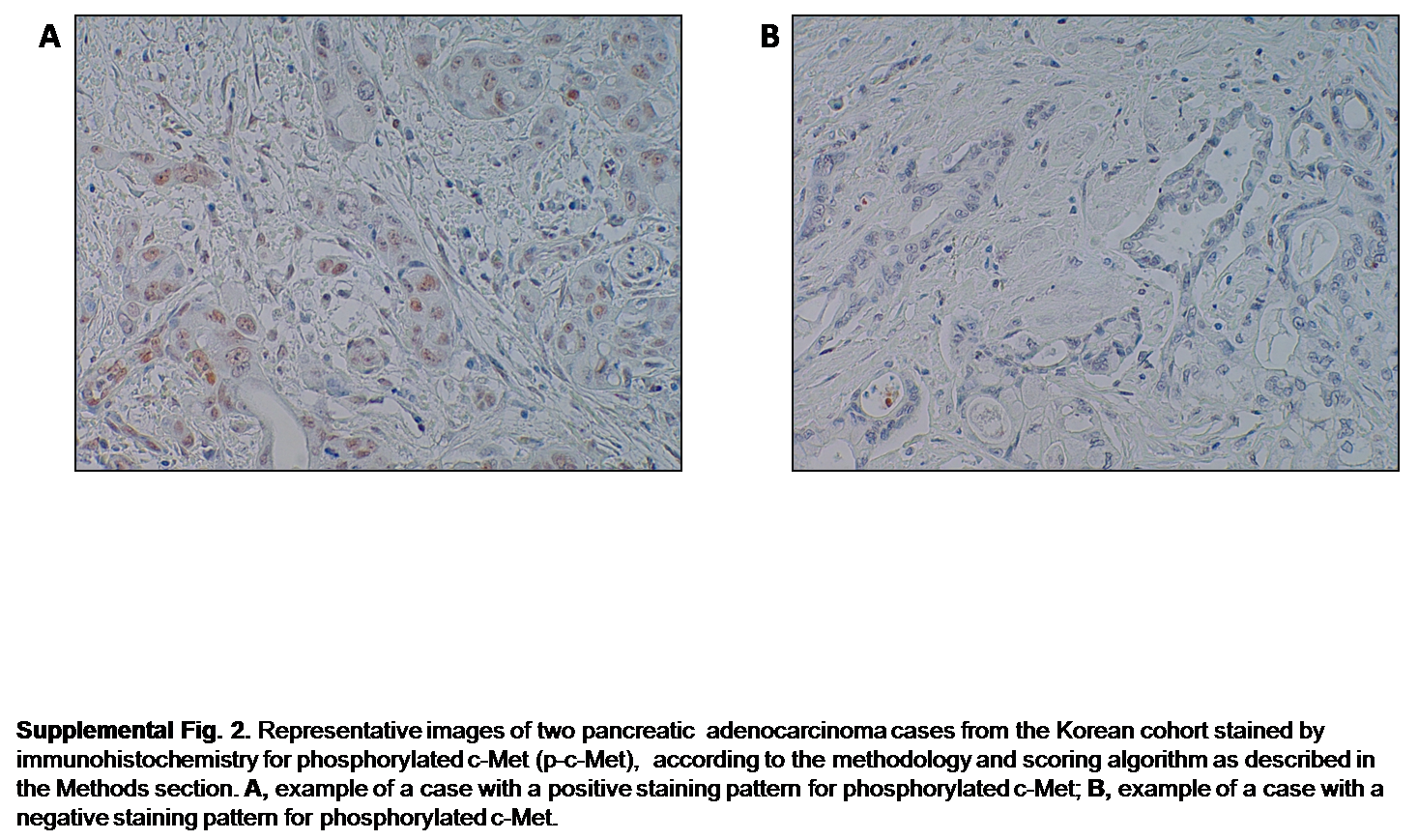

Supplement: Figure S2 — Representative images of two pancreatic adenocarcinoma cases from the Korean cohort stained by immunohistochemistry for phosphorylated c-Met (p-c-Met), according to the methodology and scoring algorithm as described in the Methods section. A, example of a case with a positive staining pattern for phosphorylated c-Met; B, example of a case with a negative staining pattern for phosphorylated c-Met. (3.82 MB TIF) [file pone.0010630.s002.tif]

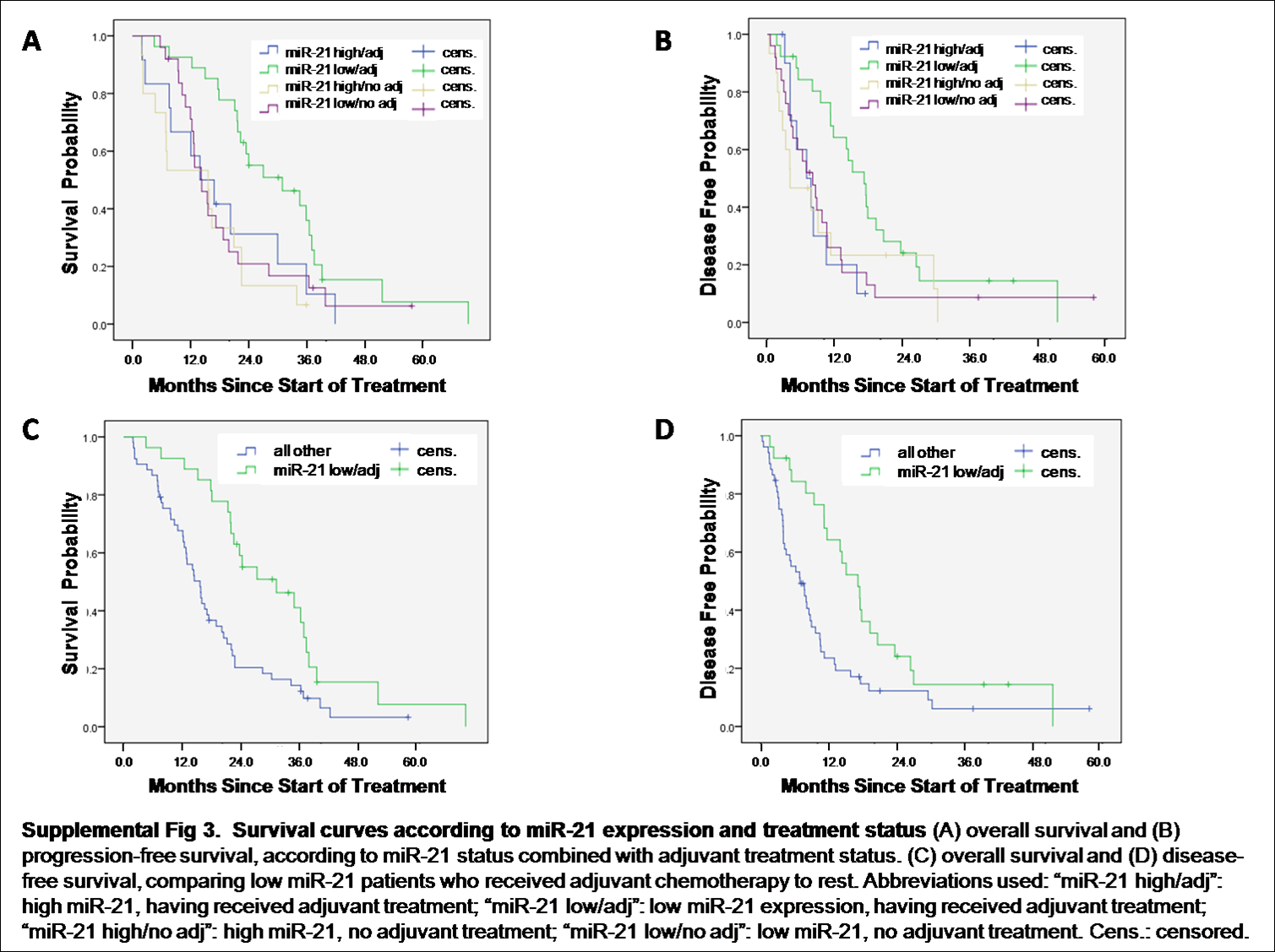

Supplement: Figure S3 — Survival curves according to miR-21 expression and treatment status (A) overall survival and (B) progression-free survival, according to miR-21 status combined with adjuvant treatment status. (C) overall survival and (D) disease-free survival, comparing low miR-21 patients who received adjuvant chemotherapy to rest. Abbreviations used: “miR-21 high/adj”: high miR-21, having received adjuvant treatment; “miR-21 low/adj”: low miR-21 expression, having received adjuvant treatment; “miR-21 high/no adj”: high miR-21, no adjuvant treatment; “miR-21 low/no adj”: low miR-21, no adjuvant treatment. Cens.: censored. (5.10 MB TIF) [file pone.0010630.s003.tif]

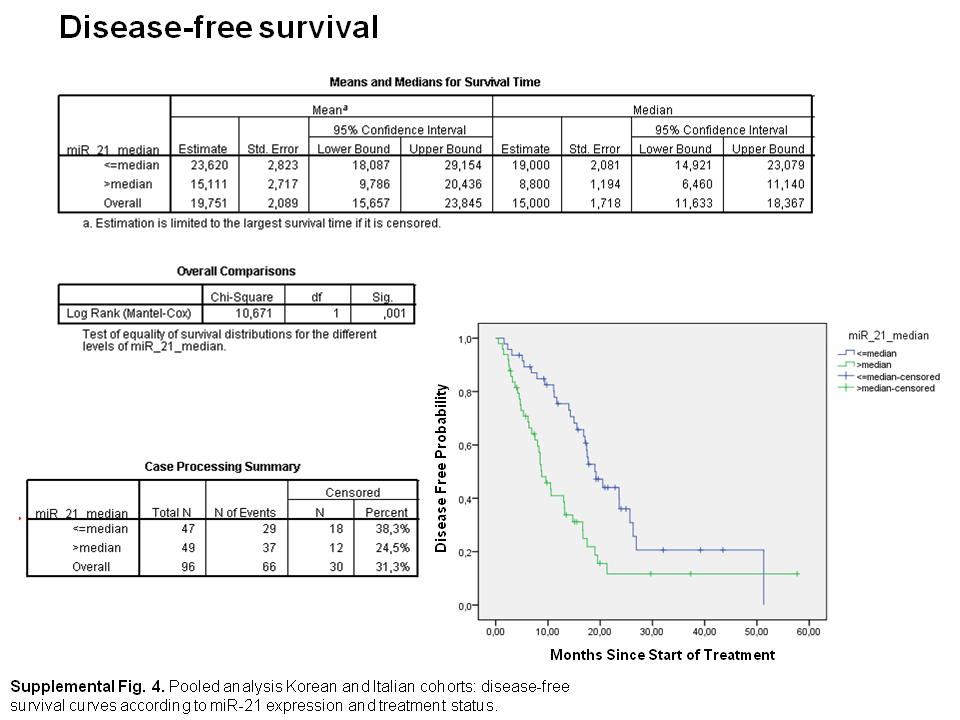

Supplement: Figure S4 — Pooled analysis Korean and Italian cohorts: disease-free survival curves according to miR-21 expression and treatment status. (0.19 MB TIF) [file pone.0010630.s004.tif]

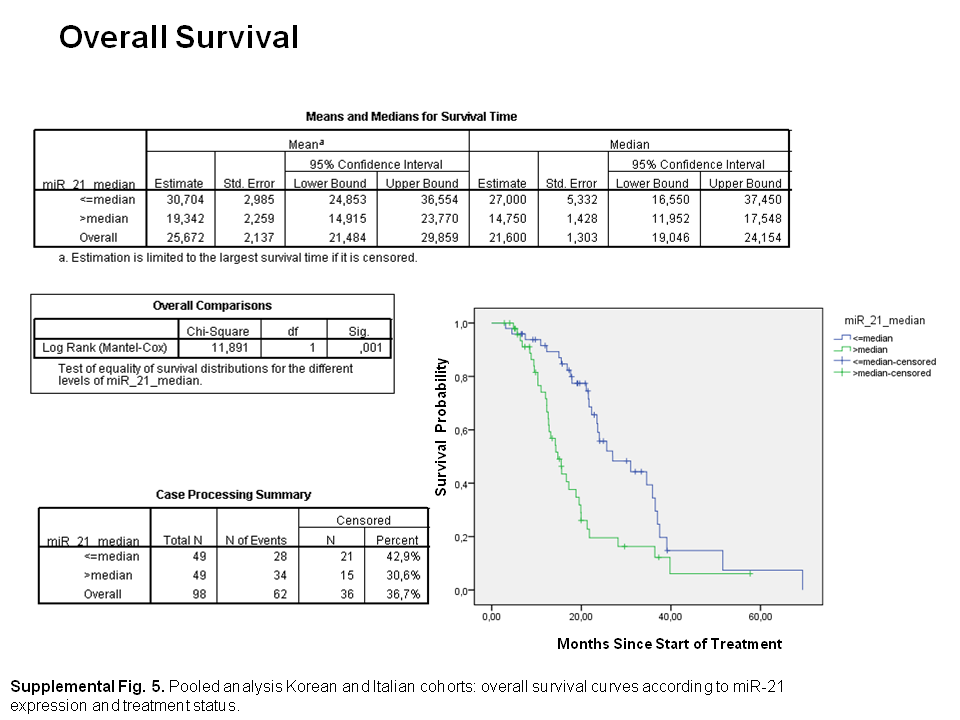

Supplement: Figure S5 — Pooled analysis Korean and Italian cohorts: overall survival curves according to miR-21 expression and treatment status. (0.19 MB TIF) [file pone.0010630.s005.tif]
