# Supplementary material for: Identification of MicroRNA-21 as a Biomarker for Chemoresistance and Clinical Outcome Following Adjuvant Therapy in Resectable Pancreatic Cancer
Source: PLoS One. 2010 May 14;5(5):e10630. doi: 10.1371/journal.pone.0010630 (PMC2871055; doi:10.1371/journal.pone.0010630)
Supplement: Table S1 — Clinicopathological and biological factors analyzed. (0.05 MB DOC) [file pone.0010630.s006.doc]

| **Supplemental Table 1.** Clinicopathological and  biological factors analyzed | |
| --- | --- |
| **Clinicopathological**  **factors** | ***Proteins (IHC)*** |
|  | Amphiregulin |
| Sex | Epiregulin |
| Age | Ron β |
| pAJCC stage | HGF |
| Tumor size (pT) | CXCR3 |
| pN stage | CXCR4 |
| Differentiation grade | E-cadherin |
| Angiolymphatic invasion | RRM1 |
| Venous invasion | ERCC1 |
| Perineural invasion | TS |
| **Biological factors** | EGFR |
| ***MicroRNAs (RT-PCR)*** | IGF-1R |
| miR-21 | Neurophilin |
| miR-29b | VEGF |
| miR-34a | c-MET / p-cMET |
| miR-34b | MMP2 |
| miR-34c | MMP7 |
| miR-155 | MMP9 |
| let-7a-2 | TIMP3 |

Abbreviations: Chemokine (C-X-C motif) receptor 3 (CXCR3), chemokine (C-X-C motif) receptor 4 (CXCR4), epidermal growth factor receptor (EGFR), excision repair cross-complementation group 1 (ERCC1), hepatocyte growth factor (HGF), insulin-like growth factor 1 receptor beta (IGF-1R), matrix metalloproteinase-2 (MMP2), matrix metalloproteinase-7 (MMP7), matrix metalloproteinase-9 (MMP9), ribonucleotide reductase subunit M1 (RRM1), thymidylate synthase (TS), tissue inhibitor of metalloproteinase*-*3 (TIMP3) and vascular endothelial growth factor (VEGF)
