# Supplementary material for: Identification of MicroRNA-21 as a Biomarker for Chemoresistance and Clinical Outcome Following Adjuvant Therapy in Resectable Pancreatic Cancer
Source: PLoS One. 2010 May 14;5(5):e10630. doi: 10.1371/journal.pone.0010630 (PMC2871055; doi:10.1371/journal.pone.0010630)
Supplement: Table S2 — Adjuvant therapy regimens. (0.05 MB DOC) [file pone.0010630.s007.doc]

| **Supplemental Table 2.** Adjuvant therapy regimens | | |
| --- | --- | --- |
| **Cohort** | **Regimen** | **Patients treated** |
| **Korean cohort***  **Adjuvant chemotherapy**  **n = 39** | gemcitabine 1000 or 1200 mg/m^2^, days 1, 8 and cisplatin 60 mg/m^2^, day 8, every 3 weeks for 2 cycles | n = 23 |
|  | gemcitabine 1000 mg/m^2^, days 1, 8 every 3 weeks for 4 cycles | n = 2 |
|  | gemcitabine 1000 mg/m^2^, days 1, 8, 15 and xeloda, 650 mg/m^2^, bid for 21 days every 4 weeks for 6 cycles | n = 2 |
|  | 5-FU 500 mg/m^2^ i.v., days 1-5 every 4 weeks for 12 cycles | n = 6 |
|  | 5-FU 500 mg/m^2^ i.v. and leucovorin 20 mg/m^2^  days 1-4, mitomycin C 8 mg/m^2^, day 1, every 4 weeks for 6 cycles | n = 1 |
|  | 5-FU 500 mg/m^2^ i.v. and leucovorin 20 mg/m^2^ days 1-4, every 4 weeks for 6 cycles | n = 4 |
|  | UFT-E 1 pkg bid for 28 days, leucovorin 15 mg 2T tid for 28 days, every 5 weeks for 6 cycles | n = 1 |
| **Korean cohort***  **Adjuvant concurrent**  **chemoradiation (CCRT)**  **n = 48** | 50.4 Gy in 28 fractions, gemcitabine weekly 300 mg/m^2^ | n = 18 |
|  | 50.4 Gy in 28 fractions, xeloda 650 mg/m^2^ bid during radiotherapy | n = 2 |
|  | 40 Gy in 20 fractions or 50.4 Gy in 28 fractions, 5-FU 500 mg/m^2^ i.v. days 1-3, every 3 weeks for 2 cycles | n = 26 |
|  | Regimen unknown | n = 2 |
| **Italian cohort****  **Adjuvant concurrent chemoradiation**  **n = 45** | gemcitabine 1000 mg/m^2^/day on days 1, 8 and 15 every 4 weeks for two cycles, followed by gemcitabine 300 mg/m^2^ weekly plus concomitant radiation therapy up to a total of 45 Gy | n = 45 |

***** Of 82 Korean patients, 52 patients received adjuvant therapy, either chemotherapy, concurrent chemo-radioterapy (CCRT), or a combination of both. Treatment started within 8 weeks following surgery. In total, 39 patients received adjuvant chemotherapy and 48 patients received CCRT. Twenty-seven patients did not receive adjuvant therapy and of 3 patients adjuvant treatment status is unknown

**Of 45 Italian patients, all received adjuvant gemcitabine chemotherapy followed by CCRT.
