# Supplementary material for: Identification of MicroRNA-21 as a Biomarker for Chemoresistance and Clinical Outcome Following Adjuvant Therapy in Resectable Pancreatic Cancer
Source: PLoS One. 2010 May 14;5(5):e10630. doi: 10.1371/journal.pone.0010630 (PMC2871055; doi:10.1371/journal.pone.0010630)
Supplement: Table S3 — Antibodies used for immunohistochemistry. (0.04 MB DOC) [file pone.0010630.s008.doc]

| **Supplemental Table 3.** Antibodies used for immunohistochemistry | |
| --- | --- |
| **Supplier** | **Antibody** |
| Abcam, Cambridge, MA | VEGF (11B5)  MMP2 (CA-4001/ CA719E3C)  MMP7 (ab4044)  MMP9 (whole molecule)  TIMP3  EGFR  ERCC1 |
| Santa Cruz Biotechnology, Santa Cruz, CA | neurophilin (A-12)  IGF-1R (3G5C1)  Ron β (C-20)  c-Met (C-28) |
| R&D, Minneapolis, MN | CXCR4 (clone 44716)  amphiregulin (polyclonal)  epiregulin (polyclonal)  HGF (polyclonal) |
| Millipore, Billerica, MA | TS  CXCR3 (polyclonal) |
| Proteintech, Chicago, IL | RRM1 |
| Invitrogen, Carlsbad, CA | p-c-Met |
| BD biosciences, Franklin Lakes, NJ | E-cadherin |

Abbreviations: Chemokine (C-X-C motif) receptor 3 (CXCR3), chemokine (C-X-C motif) receptor 4 (CXCR4), epidermal growth factor receptor (EGFR), excision repair cross-complementation group 1 (ERCC1), hepatocyte growth factor (HGF), insulin-like growth factor 1 receptor beta (IGF-1R), matrix metalloproteinase-2 (MMP2), matrix metalloproteinase-7 (MMP7), matrix metalloproteinase-9 (MMP9), ribonucleotide reductase subunit M1 (RRM1), thymidylate synthase (TS), tissue inhibitor of metalloproteinase*-*3 (TIMP3) and vascular endothelial growth factor (VEGF)
