# Supplementary material for: Identification of MicroRNA-21 as a Biomarker for Chemoresistance and Clinical Outcome Following Adjuvant Therapy in Resectable Pancreatic Cancer
Source: PLoS One. 2010 May 14;5(5):e10630. doi: 10.1371/journal.pone.0010630 (PMC2871055; doi:10.1371/journal.pone.0010630)
Supplement: Table S4 — Univariate analysis Korean cohort. (0.07 MB DOC) [file pone.0010630.s009.doc]

| **Supplemental Table 4.** Univariate analysis Korean cohort | | | | |
| --- | --- | --- | --- | --- |
| **Endpoint** | **Parameter** | **All patients**  **p-value** | **Not adjuvant treated**  **p-value** | **Adjuvant treated**  **p-value** |
| **Overall**  **survival**  **(OS)** | miR-21 status  - negative vs. positive | 0.013* | 0.49 | 0.016 |
|  | Treatment status  - not adjuvant vs. adjuvant | 0.0166 | NA | NA |
|  | p-AJCC stage  - IIa vs. IIB | 0.077 | 0.71 | 0.13 |
|  | Angiolymphatic invasion  - negative vs. positive | 0.0244 | 0.0084 | 0.22 |
|  | TIMP3 status  - negative vs. positive | 0.061 | 0.91 | 0.0246 |
|  | CXCR3 status  - negative vs. positive | 0.0191 | 0.811 | 0.0039 |
|  | Adjuvant treated, miR-21  - negative patients vs. rest | 0.0020 | NA | NA |
|  | Adjuvant treated, pN  - negative patients vs. rest | 0.016 | NA | NA |
|  | Adjuvant treated, CXCR3  - negative patients vs. rest | 0.001 | NA | NA |
|  | Adjuvant treated, TIMP3  - negative patients vs. rest | 0.0047 | NA | NA |
| **Disease-free**  **survival**  **(DFS)** | miR-21 status  - negative vs. positive | 0.039* | 0.93 | 0.02 |
|  | miR-34a status  - negative vs. positive | 0.014* | 0.0018 | 0.17 |
|  | Treatment status  - not adjuvant vs. adjuvant | 0.085 | NA | NA |
|  | Angiolymphatic invasion  - negative vs. positive | 0.066 | 0.023 | 0.37 |
|  | Amphiregulin status  - negative vs. positive | 0.06 | 0.10 | 0.91 |
|  | pN status  - negative vs. positive | 0.082 | 0.85 | 0.03 |
|  | p-c-MET status  - negative vs. positive | 0.033 | 0.089 | 0.031 |
|  | Adjuvant treated, miR-21  - negative vs. rest | 0.0095 | NA | NA |
|  | No adjuvant treated, miR-34a  - positive vs. rest | <0.0001 | NA | NA |
|  | Adjuvant treated, pN  - negative vs. rest | 0.0145 | NA | NA |
|  | Not adjuvant treated, angiolymphatic  - positive vs. rest | 0.0002 | NA | NA |
|  | Not adjuvant treated, amphiregulin  - negative vs. rest | 0.0012 | NA | NA |

*Unadjusted p-values; adjusted p-values for miR-21 status (OS), miR-21 status (DFS) and miR-34a status (DFS): 0.038, 0.12 and 0.042, respectively; NA: not applicable
