# Supplementary material for: Identification of MicroRNA-21 as a Biomarker for Chemoresistance and Clinical Outcome Following Adjuvant Therapy in Resectable Pancreatic Cancer
Source: PLoS One. 2010 May 14;5(5):e10630. doi: 10.1371/journal.pone.0010630 (PMC2871055; doi:10.1371/journal.pone.0010630)
Supplement: Table S5 — Korean cohort: clinicopathological covariates according to treatment status. (0.07 MB DOC) [file pone.0010630.s010.doc]

| **Supplemental Table 5.** Korean cohort: clinicopathological covariates  according to treatment status | | | | | | | | |
| --- | --- | --- | --- | --- | --- | --- | --- | --- |
| **Parameter** | | **No adjuvant** | | **Adjuvant** | | **Total** | | **p-values** |
|  |  | **n** | **%** | **n** | **%** | **n** | **%** |  |
| **SEX** | Male | 16 | 59% | 37 | 71% | 53 | 67% | 0.32 |
|  | Female | 11 | 41% | 15 | 29% | 26 | 33% |  |
| **AGE** | <55 years | 1 | 4% | 9 | 17% | 10 | 13% | 0.02 |
|  | 55-64 years | 10 | 37% | 28 | 54% | 38 | 48% |  |
|  | >64 years | 16 | 59% | 15 | 29% | 31 | 39% |  |
| **p-AJCC Stage** | IIa | 8 | 30% | 22 | 42% | 30 | 38% | 0.46 |
|  | IIb | 18 | 67% | 30 | 58% | 48 | 61% |  |
|  | III | 1 | 4% | 0 | 0% | 1 | 1% |  |
| **Tumor size (pT)** | <15mm | 2 | 7% | 1 | 2% | 3 | 16% | 0.39 |
|  | 15-20mm | 2 | 7% | 7 | 13% | 9 | 11% |  |
|  | >20mm | 23 | 85% | 44 | 85% | 67 | 85% |  |
| **pN stage** | 0 | 8 | 30% | 20 | 38% | 28 | 35% | 0.46 |
|  | 1 | 19 | 70% | 30 | 58% | 49 | 62% |  |
|  | unknown | 0 | 0% | 2 | 4% | 2 | 3% |  |
| **Differentiation grade** | Well | 3 | 11% | 3 | 6% | 6 | 8% | 0.59 |
|  | Moderate | 20 | 74% | 44 | 85% | 64 | 81% |  |
|  | Poor/  undifferentiated | 3 | 11% | 5 | 10% | 8 | 10% |  |
|  | unknown | 1 | 4% | 0 | 0% | 1 | 1% |  |
| **Angiolymphatic invasion** | No | 13 | 48% | 27 | 52% | 40 | 51% | 0.81 |
|  | Yes | 14 | 52% | 25 | 48% | 39 | 49% |  |
| **Venous invasion** | No | 20 | 74% | 42 | 81% | 62 | 78% | 0.57 |
|  | Yes | 7 | 26% | 10 | 19% | 17 | 22% |  |
| **Perineural invasion** | No | 6 | 22% | 15 | 29% | 21 | 27% | 0.60 |
|  | Yes | 21 | 78% | 37 | 71% | 58 | 73% |  |
