# Supplementary material for: Identification of MicroRNA-21 as a Biomarker for Chemoresistance and Clinical Outcome Following Adjuvant Therapy in Resectable Pancreatic Cancer
Source: PLoS One. 2010 May 14;5(5):e10630. doi: 10.1371/journal.pone.0010630 (PMC2871055; doi:10.1371/journal.pone.0010630)
Supplement: Table S6 — Korean cohort: immunohistochemistry covariates according to treatment status. (0.10 MB DOC) [file pone.0010630.s011.doc]

| **Supplemental Table 6.** Korean cohort: immunohistochemistry covariates  according to treatment status | | | | | | | | |
| --- | --- | --- | --- | --- | --- | --- | --- | --- |
| **Proteins** | | **No adjuvant** | | **Adjuvant** | | **Total** | | **p-value** |
|  |  | **n** | **%** | **n** | **%** | **n** | **%** |  |
| **Amphiregulin** | Negative | 16 | 64% | 11 | 22% | 27 | 36% | 0.0007 |
|  | Positive | 9 | 36% | 40 | 78% | 49 | 64% |  |
| **Epiregulin** | Negative | 9 | 38% | 13 | 28% | 22 | 31% | 0.43 |
|  | Positive | 15 | 63% | 34 | 72% | 49 | 69% |  |
| **Ron β** | Negative | 11 | 46% | 7 | 14% | 18 | 24% | 0.004 |
|  | Positive | 13 | 54% | 44 | 86% | 57 | 76% |  |
| **HGF** | Negative | 9 | 36% | 20 | 39% | 29 | 38% | 1.00 |
|  | Positive | 16 | 64% | 31 | 61% | 47 | 62% |  |
| **CXCR3** | Negative | 21 | 88% | 43 | 84% | 64 | 85% | 1.00 |
|  | Positive | 3 | 13% | 8 | 16% | 11 | 15% |  |
| **CXCR4** | Negative | 4 | 17% | 7 | 14% | 11 | 15% | 0.74 |
|  | Positive | 20 | 83% | 43 | 86% | 63 | 85% |  |
| **E-cadherin** | Negative | 1 | 4% | 0 | 0% | 1 | 1% | 0.32 |
|  | Positive | 23 | 96% | 51 | 100% | 74 | 99% |  |
| **RRM1** | Negative | 19 | 79% | 43 | 84% | 62 | 83% | 0.74 |
|  | Positive | 5 | 21% | 8 | 16% | 13 | 17% |  |
| **ERCC1** | Negative | 14 | 61% | 32 | 65% | 46 | 64% | 0.79 |
|  | Positive | 9 | 39% | 17 | 35% | 26 | 36% |  |
| **ERCC1 (H-score)** | Negative | 7 | 30% | 11 | 22% | 18 | 25% | 0.56 |
|  | Positive | 16 | 70% | 38 | 78% | 54 | 75% |  |
| **TS** | Negative | 23 | 96% | 48 | 94% | 71 | 95% | 1.00 |
|  | Positive | 1 | 4% | 3 | 6% | 4 | 5% |  |
| **EGFR** | Negative | 19 | 79% | 40 | 77% | 59 | 78% | 1.00 |
|  | Positive | 5 | 21% | 12 | 23% | 17 | 22% |  |
| **IGF-1R** | Negative | 20 | 80% | 37 | 93% | 57 | 76% | 0.78 |
|  | Positive | 5 | 20% | 13 | 8% | 18 | 24% |  |
| **Neurophilin** | Negative | 10 | 38% | 17 | 33% | 27 | 35% | 0.80 |
|  | Positive | 16 | 62% | 34 | 67% | 50 | 65% |  |
| **VEGF** | Negative | 9 | 45% | 16 | 23% | 25 | 33% | 0.61 |
|  | Positive | 15 | 55% | 35 | 78% | 50 | 67% |  |
| **c-MET** | Negative | 16 | 67% | 40 | 82% | 56 | 77% | 0.24 |
|  | Positive | 8 | 33% | 9 | 18% | 17 | 23% |  |
| **phosporylated-c-MET** | Negative | 17 | 71% | 23 | 47% | 40 | 55% | 0.08 |
|  | Positive | 7 | 29% | 26 | 53% | 33 | 45% |  |
| **MMP2** | Negative | 10 | 42% | 16 | 31% | 26 | 35% | 0.44 |
|  | Positive | 14 | 58% | 35 | 69% | 49 | 65% |  |
| **MMP7** | Negative | 7 | 29% | 14 | 27% | 21 | 28% | 1.00 |
|  | Positive | 17 | 71% | 37 | 73% | 54 | 72% |  |
| **MMP9** | Negative | 3 | 12% | 4 | 8% | 7 | 9% | 0.68 |
|  | Positive | 23 | 88% | 47 | 92% | 70 | 91% |  |
| **TIMP3** | Negative | 9 | 38% | 19 | 37% | 28 | 37% | 1.00 |
|  | Positive | 15 | 63% | 32 | 63% | 47 | 63% |  |

Abbreviations: Chemokine (C-X-C motif) receptor 3 (CXCR3), chemokine (C-X-C motif) receptor 4 (CXCR4), epidermal growth factor receptor (EGFR), excision repair cross-complementation group 1 (ERCC1), hepatocyte growth factor (HGF), insulin-like growth factor 1 receptor beta (IGF-1R), matrix metalloproteinase-2 (MMP2), matrix metalloproteinase-7 (MMP7), matrix metalloproteinase-9 (MMP9), ribonucleotide reductase subunit M1 (RRM1), thymidylate synthase (TS), tissue inhibitor of metalloproteinase*-*3 (TIMP3) and vascular endothelial growth factor (VEGF)
