# Supplementary material for: Identification of MicroRNA-21 as a Biomarker for Chemoresistance and Clinical Outcome Following Adjuvant Therapy in Resectable Pancreatic Cancer
Source: PLoS One. 2010 May 14;5(5):e10630. doi: 10.1371/journal.pone.0010630 (PMC2871055; doi:10.1371/journal.pone.0010630)
Supplement: Table S7 — Korean cohort: univariate analysis in adjuvant treated patients. (0.04 MB DOC) [file pone.0010630.s012.doc]

| **Supplemental Table 7.** Korean cohort: univariate analysis in adjuvant treated patients | | |
| --- | --- | --- |
| **Endpoint** | **Parameter** | **p-value** |
| **Overall**  **survival**  **(OS)** | miR-21 status  - negative vs.positive | 0.016 |
|  | CXCR3 status  - negative vs. positive | 0.0039 |
|  | AJCC stage  - IIa vs. IIB | 0.074 |
| **Disease-free**  **survival**  **(DFS)** | miR-21 status  - negative vs. positive | 0.02 |
|  | pN status  - negative vs. positive | 0.03 |
