# Supplementary material for: Identification of MicroRNA-21 as a Biomarker for Chemoresistance and Clinical Outcome Following Adjuvant Therapy in Resectable Pancreatic Cancer
Source: PLoS One. 2010 May 14;5(5):e10630. doi: 10.1371/journal.pone.0010630 (PMC2871055; doi:10.1371/journal.pone.0010630)
Supplement: Table S8 — Korean cohort: univariate analysis in not adjuvant treated patients Korean cohort. (0.04 MB DOC) [file pone.0010630.s013.doc]

| **Supplemental Table 8.** Korean cohort: univariate analysis  in not adjuvant treated patients Korean cohort | | |
| --- | --- | --- |
| **Endpoint** | **Parameter** | **p-value** |
| **Overall**  **survival**  **(OS)** | Angiolymphatic invasion  - negative vs. positive | 0.0084 |
| **Disease-free**  **survival**  **(DFS)** | miR-34a status  - negative vs. positive | 0.0018 |
