# Supplementary material for: Identification of MicroRNA-21 as a Biomarker for Chemoresistance and Clinical Outcome Following Adjuvant Therapy in Resectable Pancreatic Cancer
Source: PLoS One. 2010 May 14;5(5):e10630. doi: 10.1371/journal.pone.0010630 (PMC2871055; doi:10.1371/journal.pone.0010630)
Supplement: Table S9 — Korean cohort: association miR-21 and immunohistochemistry covariates. (0.09 MB DOC) [file pone.0010630.s014.doc]

| **Supplemental Table 9.** Korean cohort: association miR-21  and immunohistochemistry covariates | | | | | | | | |
| --- | --- | --- | --- | --- | --- | --- | --- | --- |
| **Korean Cohort** | | **Low** | | **High** | | **Total** | | **p-value** |
|  |  | **n** | **%** | **n** | **%** | **n** | **%** |  |
| **Amphiregulin** | Negative | 13 | 33% | 15 | 38% | 28 | 35% | 0.64 |
|  | Positive | 27 | 68% | 24 | 62% | 51 | 65% |  |
| **Epiregulin** | Negative | 11 | 31% | 11 | 30% | 22 | 30% | 1.00 |
|  | Positive | 25 | 69% | 26 | 70% | 51 | 70% |  |
| **Ron β** | Negative | 10 | 26% | 8 | 21% | 18 | 23% | 0.79 |
|  | Positive | 29 | 74% | 31 | 79% | 60 | 77% |  |
| **HGF** | Negative | 11 | 28% | 19 | 49% | 30 | 38% | 0.07 |
|  | Positive | 29 | 73% | 20 | 51% | 49 | 62% |  |
| **CXCR3** | Negative | 33 | 85% | 34 | 87% | 67 | 86% | 1.00 |
|  | Positive | 6 | 15% | 5 | 13% | 11 | 14% |  |
| **CXCR4** | Negative | 6 | 16% | 6 | 15% | 12 | 16% | 1.00 |
|  | Positive | 32 | 84% | 33 | 85% | 65 | 84% |  |
| **E-cadherin** | Negative | 0 | 0% | 1 | 3% | 1 | 1% | 1.00 |
|  | Positive | 39 | 100% | 38 | 97% | 77 | 99% |  |
| **RRM1** | Negative | 29 | 76% | 35 | 88% | 64 | 82% | 0.25 |
|  | Positive | 9 | 24% | 5 | 13% | 14 | 18% |  |
| **ERCC1** | Negative | 21 | 57% | 27 | 71% | 48 | 64% | 0.23 |
|  | Positive | 16 | 43% | 11 | 29% | 27 | 36% |  |
| **ERCC1 (H-score)** | Negative | 9 | 24% | 10 | 26% | 19 | 25% | 1.00 |
|  | Positive | 28 | 76% | 28 | 74% | 56 | 75% |  |
| **TS** | Negative | 37 | 95% | 36 | 92% | 73 | 94% | 1.00 |
|  | Positive | 2 | 5% | 3 | 8% | 5 | 6% |  |
| **EGFR** | Negative | 30 | 77% | 32 | 80% | 62 | 78% | 0.79 |
|  | Positive | 9 | 23% | 8 | 20% | 17 | 22% |  |
| **IGF-1R** | Negative | 29 | 74% | 30 | 77% | 59 | 76% | 1.00 |
|  | Positive | 10 | 26% | 9 | 23% | 19 | 24% |  |
| **Neurophilin** | Negative | 12 | 29% | 16 | 40% | 28 | 35% | 0.35 |
|  | Positive | 29 | 71% | 23 | 60% | 52 | 65% |  |
| **VEGF** | Negative | 17 | 45% | 9 | 23% | 26 | 33% | 0.05 |
|  | Positive | 21 | 55% | 31 | 78% | 52 | 67% |  |
| **c-Met** | Negative | 25 | 68% | 33 | 85% | 58 | 76% | 0.11 |
|  | Positive | 12 | 32% | 6 | 15% | 18 | 24% |  |
| **phosporylated-c-Met** | Negative | 27 | 71% | 15 | 39% | 42 | 55% | 0.01 |
|  | Positive | 11 | 29% | 23 | 61% | 34 | 45% |  |
| **MMP2** | Negative | 15 | 38% | 12 | 31% | 27 | 35% | 0.63 |
|  | Positive | 24 | 62% | 27 | 69% | 51 | 65% |  |
| **MMP7** | Negative | 9 | 24% | 13 | 33% | 22 | 28% | 0.46 |
|  | Positive | 29 | 76% | 27 | 68% | 56 | 72% |  |
| **MMP9** | Negative | 3 | 8% | 4 | 10% | 7 | 9% | 1.00 |
|  | Positive | 37 | 93% | 36 | 90% | 73 | 91% |  |
| **TIMP3** | Negative | 14 | 37% | 15 | 38% | 29 | 37% | 1.00 |
|  | Positive | 24 | 63% | 25 | 63% | 49 | 63% |  |

Abbreviations. Chemokine (C-X-C motif) receptor 3 (CXCR3), chemokine (C-X-C motif) receptor 4 (CXCR4), epidermal growth factor receptor (EGFR), excision repair cross-complementation group 1 (ERCC1), hepatocyte growth factor (HGF), insulin-like growth factor 1 receptor beta (IGF-1R), matrix metalloproteinase-2 (MMP2), matrix metalloproteinase-7 (MMP7), matrix metalloproteinase-9 (MMP9), ribonucleotide reductase subunit M1 (RRM1), thymidylate synthase (TS), tissue inhibitor of metalloproteinase*-*3 (TIMP3) and vascular endothelial growth factor (VEGF)
