# Supplementary material for: Identification of MicroRNA-21 as a Biomarker for Chemoresistance and Clinical Outcome Following Adjuvant Therapy in Resectable Pancreatic Cancer
Source: PLoS One. 2010 May 14;5(5):e10630. doi: 10.1371/journal.pone.0010630 (PMC2871055; doi:10.1371/journal.pone.0010630)
Supplement: Table S10 — Italian cohort: univariate analysis in adjuvant treated patients. (0.04 MB DOC) [file pone.0010630.s015.doc]

| **Supplemental Table 10.** Italian cohort: univariate analysis in adjuvant treated patients | | |
| --- | --- | --- |
| **Endpoint** | **parameter** | **p-value** |
| **Overall**  **survival**  **(OS)** | miR-21 status  - negative vs.positive | 0.0060 |
|  | Differentiation  - poor vs. rest | 0.065 |
| **Disease-free**  **survival**  **(DFS)** | Perineural invasion  - negative vs.positive | 0.047 |
|  | miR-21 status  - negative vs.positive | 0.0042 |
